# The protective effects of maltol on cisplatin-induced nephrotoxicity through the AMPK-mediated PI3K/Akt and p53 signaling pathways

**DOI:** 10.1038/s41598-018-34156-6

**Published:** 2018-10-29

**Authors:** Xiao-jie Mi, Jin-gang Hou, Zi Wang, Ye Han, Shen Ren, Jun-nan Hu, Chen Chen, Wei Li

**Affiliations:** 1 College of Chinese Medicinal Materials, Jilin Agricultural University, Changchun, 130118 China; 2 Intelligent Synthetic Biology Center, Daejeon, 34141 Republic of Korea; 3 School of Biomedical Sciences, Queensland Brain Institute, The University of Queensland, Brisbane, Australia

## Abstract

Cisplatin, a potent anticancer drug, is usually causing nephrotoxicity; limiting its therapeutic application and efficiency. Maltol may be used to prevent such toxic effect. The aim of this study was to investigate the underlying protective mechanisms of maltol on nephrotoxicity by cisplatin using a cisplatin-treated mouse model and a cellular toxicity model of HEK293 cells. The blood urea nitrogen (BUN), creatinine (CRE) and neutrophil gelatinase-associated lipocalin (NGAL) levels in mice were increased by cisplatin but decreased to normal ranges by maltol pretreatment (50 and 100 mg/kg) for ten days. Besides, maltol pretreatment decreased oxidative stress, lipid peroxidation and apoptosis in cisplatin-treated mice. The inhibitory action of maltol on inflammatory responses was achieved by reducing the expressions in NF-κB, IL-1β, iNOS, and TNF-α in the mice *in vivo*. Additionally, maltol restored the reduction of PI3K/Akt and mTOR levels by cisplatin through increasing AMPK expression in cisplatin-treated HEK293 cells. Maltol also suppressed the expression of Bax and caspase 3 by inhibiting the p53 activity in HEK293 cells. Overall, maltol may serve as a valuable potential drug to prevent cisplatin-induced nephrotoxicity, and the underlying molecular mechanisms of maltol action may involve intracellular AMPK/PI3K/Akt and p53 signaling pathways.

## Introduction

Cisplatin is widely used in clinical practice to treat a variety of solid tumors owing to its high efficiency and easy administration^1^. The efficacy of cisplatin is proportional to its dose. However, it has a relative narrow safety range and shows serious dose-limiting side effects including neurotoxicity, ototoxicity, nausea, vomiting, and especially nephrotoxicity^2^. Therefore, reducing renal injury in cisplatin-treated patients is in urgent need, and the pathogenesis of cisplatin should be clarified to develop a new drug to extenuate the cisplatin-caused nephrotoxicity^3,4^. To date, although the pathophysiological basis of cisplatin nephrotoxicity has been studied in recent years, the molecular mechanism of cisplatin-induced renal injury has not been clarified yet. There is evidence though that necrosis, oxidative stress, inflammation, and apoptosis may play crucial roles in cisplatin-induced nephrotoxicity^5^.

Cisplatin-induced nephrotoxicity results in severe nephropathy involving acute renal failure with histological changes of the renal tubular cells^6^. A direct result of nephrotoxicity is the loss of renal functions, including severe reductions in glomerular filtration, creatinine (CRE) clearance and corresponding increases in serum creatinine, blood urea nitrogen (BUN) and neutrophil gelatinase-associated lipocalin (NGAL)^7,8^. Connected with the cisplatin-induced loss of renal function, there was a marked decrease in body weight and an increase in kidney index in cisplatin-treated mice with increase in serum CRE, BUN and NGAL. In addition, several urinary enzymes, e.g., N-acetyl-β-D-glucosaminidase (NAG) and kidney injury molecule-1 (KIM-1) have been used as nephrotoxic biomarkers, suggesting a gastrointestinal toxicity and renal dysfunction, respectively^9,10^. Furthermore, the platinum concentrations in kidney samples of cisplatin-treated mice were significantly higher than that in normal group. Recent evidence suggests that cisplatin infiltrates into the cells leading to mitochondrial dysfunction and accumulated lipid peroxidation products in the kidney, causing the rapid generation of reactive oxygen species (ROS) and activate the oxidative metabolism system. ROS accumulation over insufficient antioxidant system results in an elevation in MDA content and attenuation of Glutathione (GSH), Superoxide dismutase (SOD) as well as the elevations of inflammation factors such as NF-κB, IL-1β, iNOS, and TNF-α^11^. The generation of proinflammatory cytokines, dysfunction of immune cells and abnormalities of the cellular PI3K/Akt pathways, collectively activate the signal pathway of apoptosis^12^. In most cases, cisplatin induces irreversible kidney damage due to excessive cell death^13^, which may be effectively alleviated by regulation of AMPK and its downstream PI3K/Akt and p53 signaling pathways^14,15^. As a key metabolic switch, AMPK is vital for cell to maintain the normal energy metabolism and redox balance^16^. Thus, cisplatin-induced kidney damage may be ameliorated by AMPK activation^17,18^. PI3K, as key downstream target of AMPK, regulates cellular features such as survival, proliferation and apoptosis^19,20^. PI3K phosphorylates Akt protein to induce proliferation, differentiation, apoptosis and migration^21,22^.

Maltol (3-hydroxy-2-methyl-4-pyrone), known as the safe and reliable flavor potentiate, food preservative and natural antioxidant, is a by-product of the maillard reaction in starch and sucrose pyrolysis^23^. It is also found in baked products as well as red ginseng root, coffee, chicory, soybeans, bread crusts, and caramelized foods^24^. Recently, maltol has been used in the fields of catalysis, cosmetic, pharmaceutical formulation as well as food chemistry^25,26^. Interestingly, it is recognized that a scavenger of ROS can be used in the treatment of anemia, tumor, nerve cell oxidative stress and diabetes-induced irreversible kidney damage^27–30^. In our previous study, we have shown that maltol attenuated acute alcohol-induced liver injury and prevented oxidative damage in mice^31^. Therefore, we hypothesize that maltol may be capable to prevent cisplatin-induced acute kidney injury.

In this study, the pretreatment of maltol effectively prevented cisplatin-induced nephrotoxicity. It was demonstrated that maltol treatment suppressed the oxidative stress, inflammation and apoptosis in cisplatin-induced renal damage, through regulation of mitochondria-dependent AMPK/PI3K/Akt and p53 signaling pathways.

## Materials and Methods

### Chemicals and Reagents

Maltol and cisplatin were manufactured by Sigma-Aldrich (St. Louis, MO, USA). DMSO, MTT were purchased from Sigma Chemicals Co. (St. Loius, MO, USA). The commercial assay kits of BUN, CRE, GSH, SOD, CAT, MDA, hematoxylin-eosin (H&E) and Periodic Acid-Schiff (PAS) kit were bought from Nanjing Jiancheng Bioengineering Research Institute (Nanjing, China). ELISA kits of mouse TNF-α and IL-1β, iNOS, NF-κB were measured using R&D systems (Minneapolis, MN, USA). Immuno-Histological Staining Kit was obtained from the Boster Biological Technology Co. Ltd (Wuhan, China). Antibodies for Bax, Bcl-2, iNOS, COX-2, caspase 3, 8, 9 and GAPDH were provided by BOSTER Biological Technology (Wuhan, China) or Cell Signaling Technology (Danvers, MA, USA). The antibodies against AMPK, phospho-AMPK (p-AMPK), Akt, phospho-Akt (p-Akt), mTOR, phospho-mTOR (p-mTOR), PI3K, phospho-PI3K (p-PI3K) and p53 were purchased from Wanlei Bio (Shenyang, China). All other chemicals and reagents, unless indicated, were provided by Beijing Chemical Factory (Beijing, China).

### Animal experiments

A total of 32 adult male ICR mice (6–8 weeks old; body weight 22–25 g) were purchased by YISI Experimental Animal Co., Ltd with Certificate of Quality No. SCXK (JI)−2016-0003 (Changchun, China). Every effort was made to reduce the number and suffering of animals. The mice were housed for 1 week before the experiment to acclimatize them to the conditions, maintained under controlled conditions (22–24 °C, 55–60% relative humidity and 12-hour light-dark cycles throughout the experiment) and fed with standard food and water ad libitum except for the day of dehydration. All experimental procedures were in accordance with the Guidelines for the Management and Use of Laboratory Animals (Ministry of Science and Technology, 2006) and approved by the Ethical Committee for Laboratory Animals of Jilin Agricultural University (Permit No.: ECLA-JLAU 2016-016).

The ICR mice were randomly divided into four groups with 8 animals each and treated for ten continuous days. The first group was used as a normal control. The second group was administered with a single injection of cisplatin (25 mg/kg i. p.). The third and the fourth groups were administered by oral gavage with maltol at a dose of 50 and 100 mg/kg, respectively once daily for ten days (7 days before and 3 days after cisplatin injection). Mice were anaesthetized with carbon dioxide at 72 h after cisplatin injection. Blood sample were collected and immediately centrifuged (4 °C, 300 g for 10 min) to gather the blood serum samples, which were subsequently stored at −20 °C for further analysis. One of the kidneys was immediately immersed in 10% neutral buffered formalin for histological analysis. For biochemical markers determination and western blot analysis, the other kidney was dissected and immediately frozen in liquid nitrogen. Relative kidney weight (%) was calculated and statistical analyzed according to the formula: relative kidney weight (%) = (kidney weight/body weight) × 100%.

### Culture of human embryonic kidney 293 (HEK293) cells

HEK293 cell line was purchased from ATCC Cell Bank. All HEK293 cells were cultured in Dulbecco′s modified eagle′s medium (DMEM) supplemented with 10% fetal bovine serum (FBS) at 37 °C under 5% CO_2._ All experiments were performed when cells were grown to 80% confluence in DMEM. HEK293 cells were seeded in 96 well plates at 1 × 10^4^ cells per well. In all experiments, cell viability was higher than 99% using trypan blue dye exclusion. The cells are incubated in different doses of cisplatin (15, 20, 25, 30 µM), cells were then washed twice with PBS and added with MTT (5 mg/mL) to determine the optimal dose of cisplatin. Then cells were treated with various concentrations of maltol 24 h prior to cisplatin (20 µM) stimulation for 24 h. Cells were then washed twice with PBS and added with MTT (5 mg/mL). After that, the cells were incubated for another 3 h at 37 °C with 5% CO_2_, the solution was then aspirated, and 150 μL dimethyl sulfoxide was added. The precipitate in each well was dissolved for 5 min and the optical density (OD) was read at 490 nm using a microplate reader. In addition, cells total protein was extracted in order to subsequent experiments.

### Parameters Analysis of Kidney Function and Renal Platinum Ion Concentration

Baseline blood samples were collected from eyeball with anaesthetized mice for analysis of biochemical levels, including BUN and CRE, and analyzed using reagents purchased from Nanjing Jiancheng Bioengineering Institute (Nanjing, China) according to the manufacturer’s instructions. Serum NGAL was measured from serum using mouse NGAL Quantikine ELISA Kit (R&D Systems) according to the manufacturer’s instruction. Nacetyl-d-glucosaminidase (NAG) and kidney injury molecule-1 (KIM-1) were purchased from Shionogi & Co., Ltd., (Osaka, Japan).

In addition, parts of the renal cortical tissue were dried overnight and the dry weight was recorded. The samples were then dissolved with equal volumes of 30% (w/v) H_2_O_2_ and 70% (w/v) nitric acid, and the clear solution was diluted with ultrapure water (1:3). Renal platinum ion concentration was analyzed using inductively coupled plasma optical emission spectrometer (Optima 2100 DV, PerkinElmer, UK) with sample-based standards.

### Analysis of Renal Oxidative Stress Indicators

Right renal tissues of the mice in appropriate size were placed in normal saline and homogenized in a ratio of 1: 9 (g): (mL) in ice bath. The supernatant was removed after 15 min of centrifugation at 3000 g under 4 °C. Finally, the SOD, MDA and GSH levels of renal tissues were determined respectively using commercial kits according to the manufacturer’s instructions.

### Determination of Cytokines in renal tissues

The levels of cytokines TNF-α, IL-1β, iNOS and NF-κB in the serum were measured using commercial ELSIA kits according to the manufacturer’s protocols. In brief, the reagents, serum samples, standard solutions and corresponding antibodies were added sequentially in the EP tube according to the instructions of the kit. Finally, the OD value of each group was measured and calculated at 450 nm via an ELISA reader (Bio-Rad, California, USA) within 10 min.

### Histopathological Examinations

After sacrificed, the mice’s left renal tissue was washed with ice-cold Stroke-physiological saline solution and fixed in 10% neutral buffered formalin solution for 24 h. After routine processing, gradient alcohol dehydration and xylene permeabilization, the renal tissues were embedded in paraffin, and sectioned at 5 μm thickness, and then sections were stained with H&E and Periodic Acid-Schiff (PAS) staining, and subsequently observed under a light microscope for histopathological examination. We can observe whether there is congestion, inflammatory cell infiltration or necrosis by H&E.

The histopathological changes of the kidneys were observed under normal H&E staining and microscopic observation. The percentage of acute renal tubular necrosis was classified and scored by semi-quantitative method, such as 0 point: normal, no necrosis; 1 point: <10%; 2 point: 10–25%; 3 point: 26–75%; 4 point: >75%. Histological examination was performed by randomized selection.

### Immunohistochemistry analysis

Immunohistochemistry (IHC) of the paraffin sections was carried out according to the manufacturer’s instructions (Boster Biological Technology, Wuhan, China). Xylene and aqueous alcohol solutions were used to deparaffinize and rehydrate paraffin slides. After antigen retrieval in a citrate buffer solution for 20 min, the slides were washed 3 times with Tris-buffered saline (TBS 0.01 M, pH 7.4) at room temperature and incubated with 1% bovine serum albumin (BSA) for 1 min. The blocking serum was tapped off, and the renal sections were incubated in a humidified chamber with primary antibodies, including mouse polyclonal anti-Bax (1:400), anti-iNOS (1:200), anti-Bcl-2 (1:200), anti-NF-κB (1:200) at 4 °C overnight, followed by secondary antibody incubation for 30 min and diaminobenzidine (DAB) Chromogen for color development at room temperature. The expression of proteins was observed under light microscopy (Olympus BX-60, Tokyo, Japan). The specificity of immunohistochemical staining is established through performing the staining of blank control group (no primary antibody incubation) and antigen expression of specific site, and the positive staining was determined mainly by a brownish-yellow color in the cytoplasm or nucleus. The intensity of positive expression was analyzed using Image-Pro Plus 6.0 software for quantification of immunohistochemistry after the paraffin sections were completely dried.

### Western blotting analysis

After treatments, HEK293 cells extracts were collected and total protein concentration was determined using the BCA protein assay kit (Beyotime Biotechnology, China). Equal amounts of protein were loaded on the 12% SDS-polyacrylamide gel electrophoresis and electroblotted onto a PVDF membrane. The membranes were blocked with 5% BSA in TBS with 0.1% Tween−20 for 2 h at room temperature, and then incubated overnight at 4 °C with indicated primary antibodies, followed by secondary antibodies conjugated with horseradish peroxidase (HRP) for 1 h at room temperature. Protein-antibody complexes were detected using Emitter Coupled Logic (ECL) substrate (Pierce Chemical Co., Rockford, IL, USA). Protein band intensities were quantified using Quantity One software (Bio-Rad Laboratories, Hercules, CA, USA).

### Statistical analysis

All data were expressed as means ± standard deviation (S.D.) derived from at least three separate experiments and analyzed with a two-tailed test or a one-way analysis of variance (ANOVA) followed by Bonferroni post-test. The *p* values of less than 0.05 or 0.01 in differences between groups were considered to be significant. Statistical graphs were produced using software of GraphPad Prism 6.0.4 software (Graphpad Software, Inc, San Diego, USA).

## Results

### Effects of maltol on blood nephrotoxic biomarkers induced by cisplatin in mice

Generally, the relative index of kidney, body weight, serum CRE and BUN were recorded, measured and calculated as basic indicators of the health status and in particular renal function of mice. Obvious attenuation of weight and hoist of relative kidney index were observed in mice administrated with cisplatin. In contrast, maltol pretreatment significantly inhibited the reduction of body weight and the increase in kidney index (*p* < 0.01) (Table 1).

**Table 1:** Effects of maltol on body weights, kidney indices, and serum biochemical markers in mice.

Groups	Dosage (mg/kg)	Body weights (g)		Kidney Index (mg/g, × 100)	CRE (µmol/L)	BUN (mmol/L)	NGAL (mg/mL)
		Initial	Final				
Normal	—	31.68	32.40	1.50	40.69 ± 4.89	8.50 ± 1.12	1.27 ± 0.24
Cisplatin	—	32.34	26.86**	2.13**	162.46 ± 24.37**	36.24 ± 2.49**	3.05 ± 0.61**
Cisplatin + maltol	50	29.89	28.99	1.53^##^	32.41 ± 10.26^##^	13.38 ± 3.57^##^	1.62 ± 0.39^##^
Cisplatin + maltol	100	30.65	29.16^#^	1.48^##^	37.77 ± 10.25^##^	8.87 ± 1.08^##^	1.88 ± 0.27^##^
Maltol-alone	100	29.95	30.45	1.61	23.64 ± 9.04*	9.01 ± 1.19	1.14 ± 0.30

Values represent the mean ± S.D., *n* = 8; **p < 0.01, *p < 0.05 *vs*. normal group; ^##^*p* < 0.01, ^#^*p* < 0.05 *vs*^.^ cisplatin group.

In addition, the levels of CRE, BUN and NGAL were increased by 299.26%, 326.35% and 240.16%, respectively of that in normal group by cisplatin (Table 1). Maltol decreased the levels of CRE, BUN and NGAL by cisplatin treatment (Table 1).

### Analysis of the urinary nephrotoxic biomarkers and renal platinum ion concentration

To investigate whether maltol protected kidney injuries, we examined urinary KIM-1 and NAG levels as the sensitive early biomarkers of cisplatin-induced acute kidney injury in mice. Urinary KIM-1 and NAG levels were significantly in mice exposure to cisplatin (25 mg/kg) for 72 h. Such increase in urinary KIM-1 and NAG levels were decreased in mice administrated with maltol for 10 days (Table 2). These data implicated that maltol exerted protective effect on kidney injury biomarkers in cisplatin-induced acute kidney injury models.

**Table 2:** Effects of maltol on kidney injury biomarkers, KIM-1, NAG and renal platinum ion concentration in cisplatin-induced mice.

Groups	Dosage (mg/kg)	KIM-1 (ng/mL)	NAG (U/g)	Platinum ion (µg/g tissue)
Normal	—	1.52 ± 0.36	14.37 ± 2.89	ND
Cisplatin	—	6.28 ± 0.95**	51.86 ± 10.24**	11.26 ± 3.18**
Cisplatin + maltol	50	4.19 ± 0.66^##^	27.59 ± 5.37^##^	7.67 ± 1.80^##^
Cisplatin + maltol	100	2.26 ± 0.73^##^	19.48 ± 4.06^##^	3.05 ± 0.74^##^
Maltol-alone	100	1.41 ± 0.48	12.14 ± 4.83	ND

Values represent the mean ± S.D., *n* = 8; ND = non-detectable, **p < 0.01 *vs*. normal group; ^##^*p* < 0.01 *vs*^.^ cisplatin group.

A significant rise in platinum ion concentration was observed in the kidney tissue of mice receiving cisplatin treatment as compared to that in the normal control mice. However, maltol-treated mice had a significantly lower renal platinum level in comparison with the cisplatin alone group (Table 2).

### Analysis of Renal Oxidative Stress Indicators

**Figure 1:**
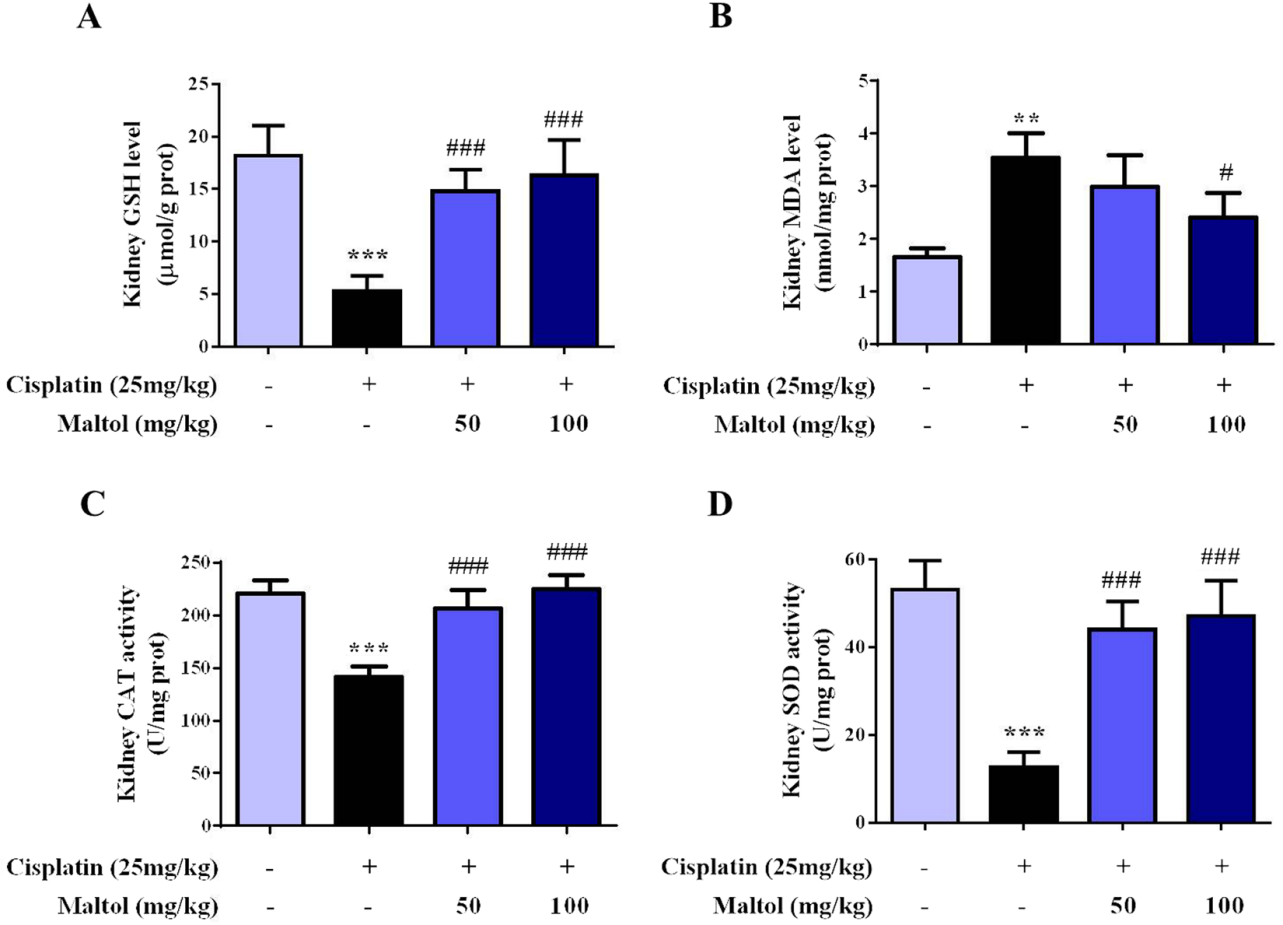
Protective effects of maltol pretreatment against cisplatin-evoked renal toxicity in mice. After cisplatin challenge, all mice were euthanized and their serums and kidneys were collected. (**A**) Kidney GSH level. (**B**) Kidney MDA level. (**C**) Kidney CAT level. (**D**) Kidney SOD level. All values were expressed as mean ± S.D. (*n* = 8 in each group). ^**^p < 0.01, ^***^p < 0.001 *vs*. normal group; ^#^*p* < 0.05, ^###^*p* < 0.001 *vs*. cisplatin group.

As oxidative stress played a key role on cisplatin-evoked renal toxicity in mice, we inspected whether maltol pretreatment could improve cisplatin-evoked oxidative stress. As indicated in Fig. 1, cisplatin dramatically attenuated renal GSH and SOD. MDA was elevated in cisplatin-treated renal tissue homogenate, indicating oxidative stress (Fig. 1, *p* < 0.001). In contrast, supplementation with maltol improved the attenuation of GSH and attenuated the level of MDA, which rehabilitated the antioxidant capacity as elaborated by the improvement of SOD and CAT levels significantly. (Fig. 1, *p* < 0.05 or *p* < 0.001).

### Maltol Ameliorates Cisplatin-Induced injury and Renal Histopathological Changes

**Figure 2:**
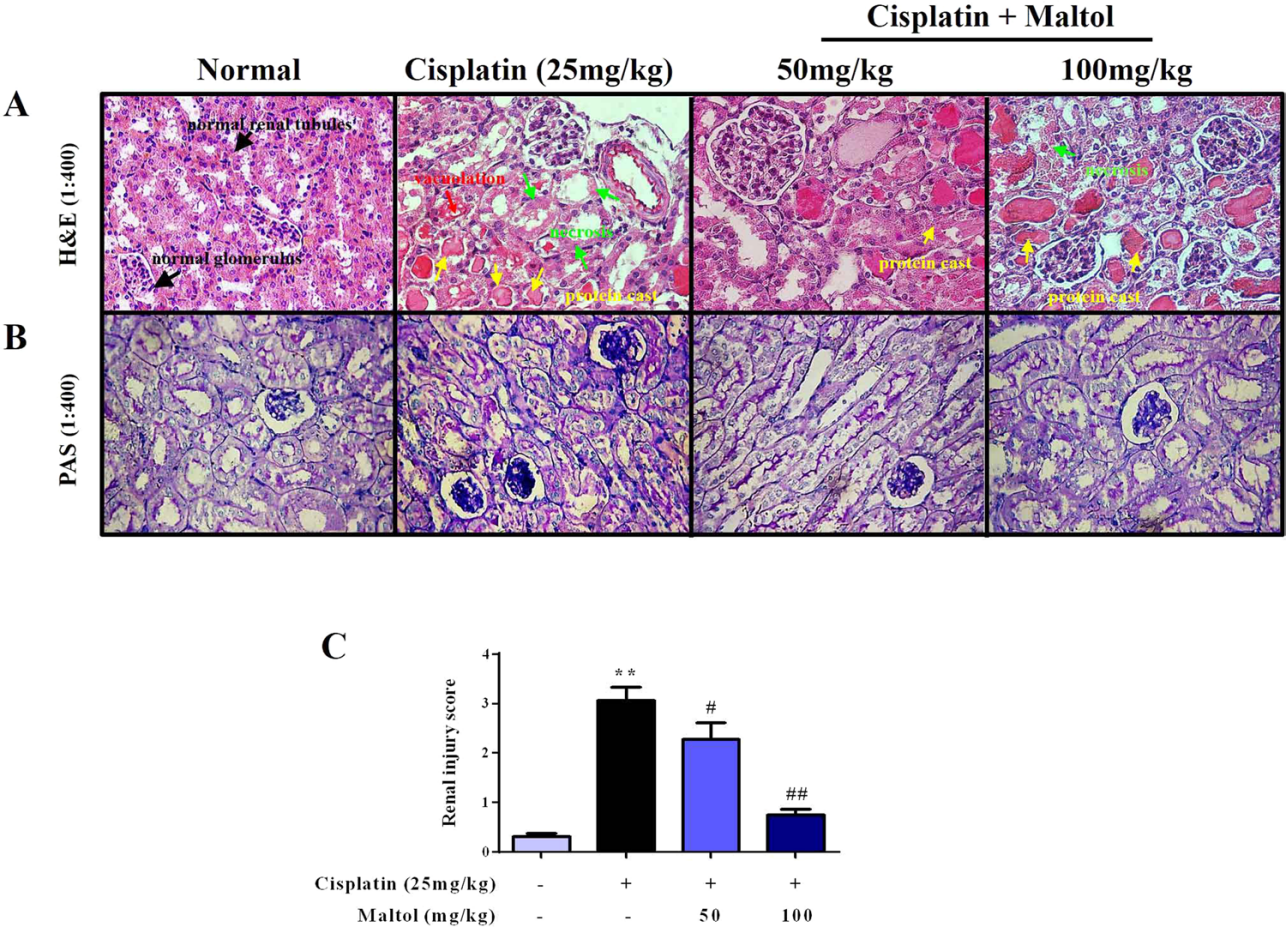
Maltol attenuated cisplatin-induced morphological changes. (**A**,**B**) H&E staining and PAS stain of kidney sections from normal group, cisplatin group, maltol (50 mg/kg) + cisplatin group and maltol (100 mg/kg) + cisplatin group. Note: The condensation of the cell necrosis (green arrow) and protein cast (yellow arrow) in cisplatin mice. Red arrows refer to vacuolization of the renal cortex (magnification ×400). The renal injury score (**C**) was determined. All values were expressed as mean ± S.D. ^**^p < 0.01 *vs*. normal group; ^#^*p* < 0.05, ^##^*p* < 0.01 *vs*. cisplatin group.

Renal histopathological changes of mice should be taken into consideration to assess the efficacy of maltol in protecting kidney from cisplatin-induced renal damage (Fig. 2). Light microscopy examination of renal tissues in normal mice revealed normal glomerulus structure and renal tubular interstitial with no evidence of cell necrosis and inflammatory infiltration (Fig. 2A). The mice treated with cisplatin showed typical damage characteristics, for example, necrosis and shedding of renal tubular epithelial cells, vacuolization of the renal cortex and inflammatory infiltrations (Fig. 2A). In comparison, maltol pretreatment reduced the number of apoptotic and infiltration of inflammatory cells in a dose-independent manner (Fig. 2A). The renal tubular necrosis score was significantly reduced compared with that in the cisplatin group, suggesting that maltol exerted potential renal protection on cisplatin-induced nephrotoxicity.

In addition, in the PAS staining (Fig. 2B), significant glycogen deposits in the renal tubules was observed in the cisplatin group, which was evidently diminished by pretreatment with maltol at low dose group, and high dose group of maltol showed less glycogen deposits in a dose dependent manner.

### Maltol Ameliorates Cisplatin-Induced Renal cells Apoptosis *in vivo*

**Figure 3:**
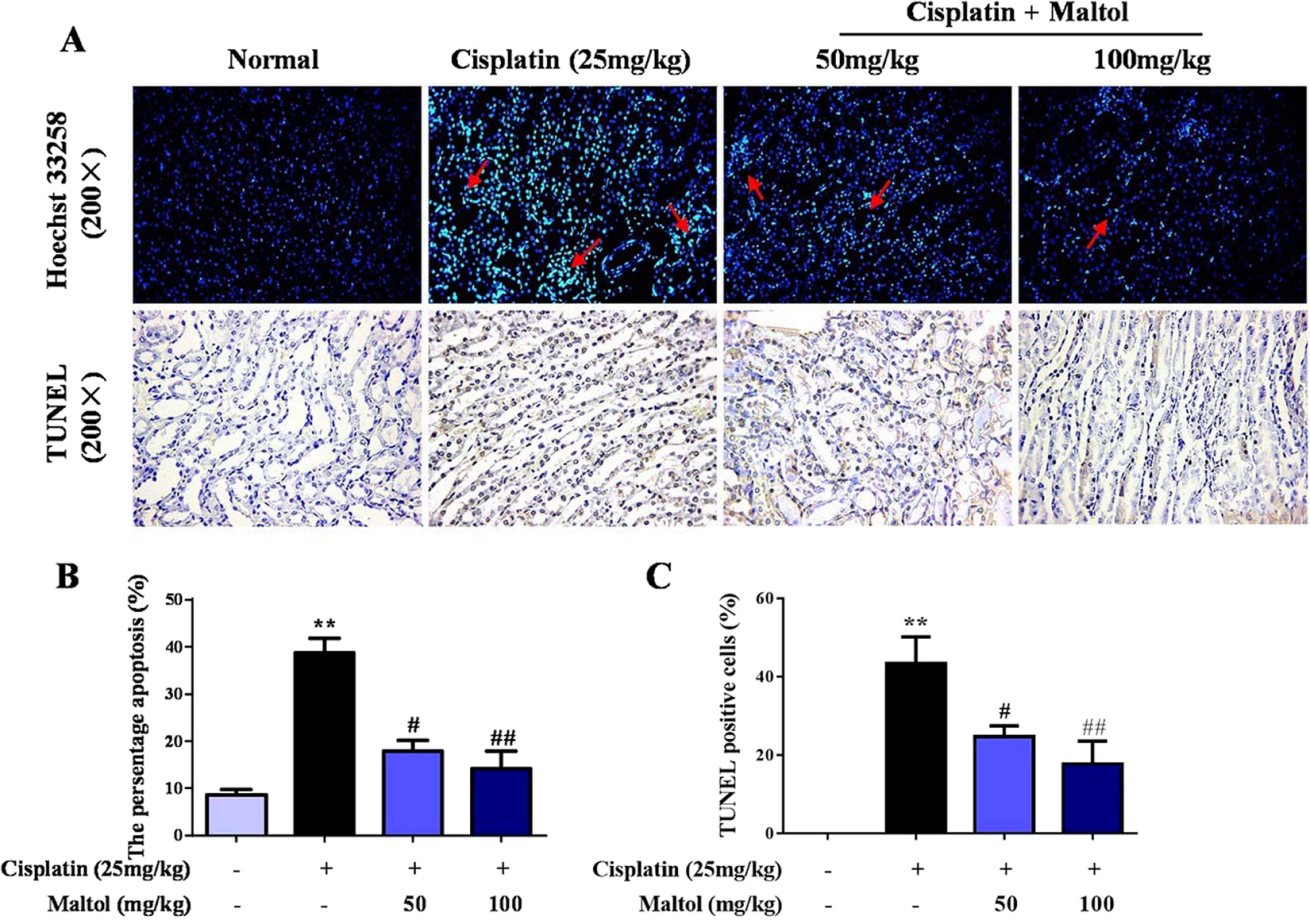
Renal tissues stained with TUNEL staining (**A**) and Hoechst 33258. (**B**) The existence of TUNEL positive cells were assessed by image analyzer. (**C**) The relative levels of fluorescence intensity were quantified. Magnification ×200. Values are expressed as mean ± S.D. ^**^p < 0.01 *vs*. normal group; ^#^*p* < 0.05, ^##^*p* < 0.01 *vs*. cisplatin group.

In order to measure the extent of apoptosis in renal tissues, Hoechst 33258 staining was performed to determine whether maltol pretreatment protected against apoptosis in cisplatin-induced acute kidney injury. As shown in Fig. 3, renal tubular epithelial cells in normal group were neatly arranged with a clear out line, and the chromatin was stained evenly and slightly. Significant nuclear fragmentation and condensation was observed in cisplatin group, indicating apoptosis of renal tubular epithelial cells (Fig. 3A). In contrast, pretreatment with maltol markedly reduced the number of apoptotic cells and inhibited renal toxicity caused by cisplatin (Fig. 3B).

To further determine whether necrosis would coexist with apoptosis in cisplatin-induced nephrotoxicity, TUNEL staining was used to confirm and quantify the apoptosis in renal cells. As shown in Fig. 3, compared to normal group, the number of TUNEL-positive tubular cells was significantly increased in cisplatin group. Pretreatment with maltol significantly decreased apoptotic cell numbers in a dose-independent manner (Fig. 3B).

**Figure 4:**
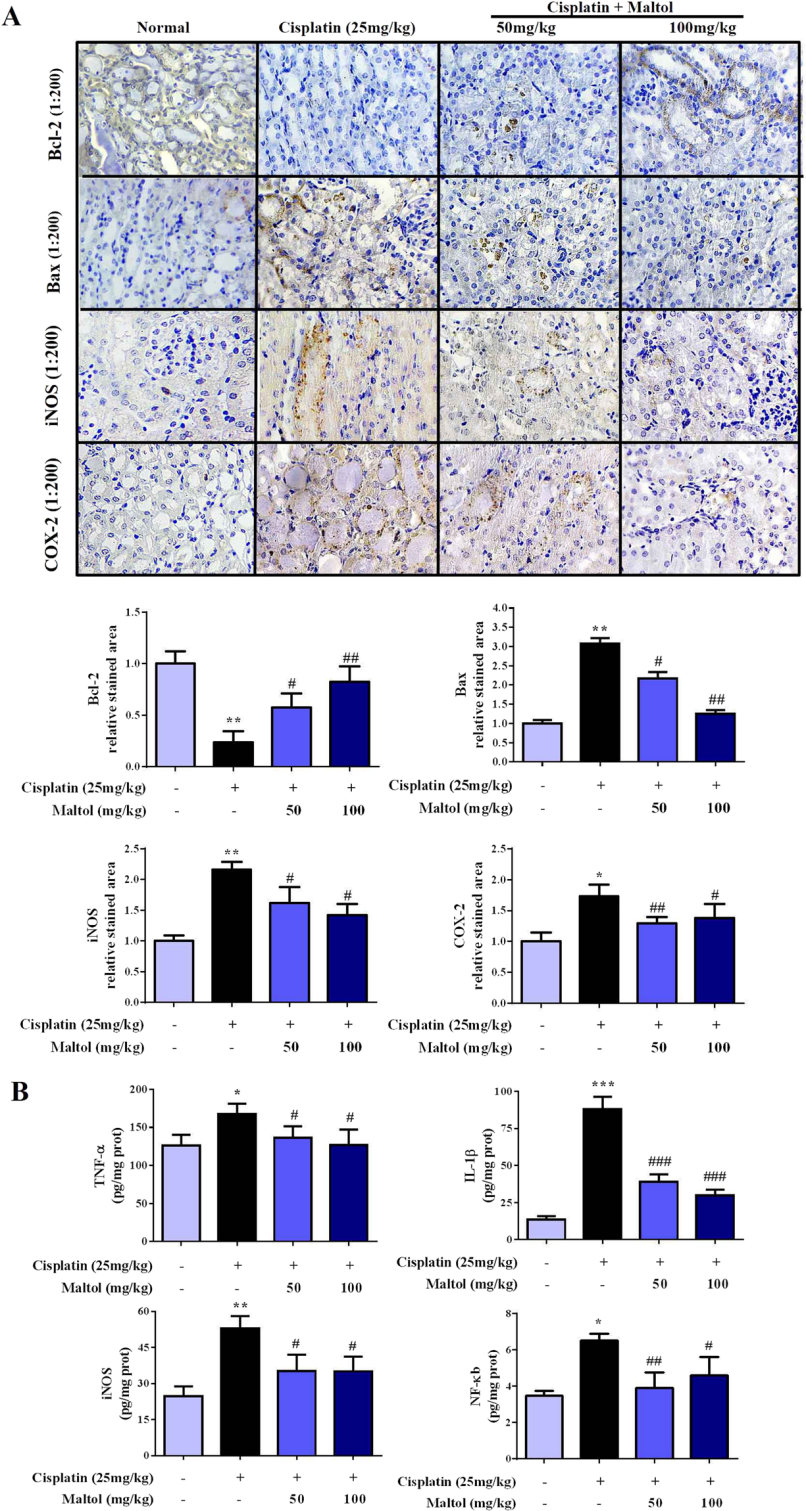
Effects of maltol on the levels of inflammation cytokines in cisplatin-induced renal toxicity. (**A**) Effects of maltol on the positive expressions of Bax, Bcl-2, iNOS and COX-2 in renal tissues were examined by IHC in renal tissues (magnification × 200), And the column chart shows stained area, semiquantitative analysis of Bax, Bcl-2, iNOS and COX-2 expression in kidneys to IHC. (**B**) Inflammation cytokines level of TNF-α, IL-1β, iNOS and NF-κB in serum of mice were measured by ELISA kits. All values were expressed as mean ± S.D. ^*^p < 0.05, ^**^p < 0.01 *vs*. normal group; ^#^*p* < 0.05, ^##^*p* < 0.01 *vs*. cisplatin group.

In order to further determine whether maltol protects against cellular apoptosis in cisplatin-induced acute kidney injury, IHC analysis was used to observe apoptotic cells in the present study. We examined the impacts of maltol on the pro-apoptotic factor Bax and anti-apoptotic factor Bcl-2. As depicted in Fig. 4A, compared with normal group, the expression of pro-apoptotic protein Bax was significant increased and the level of Bcl-2 was decreased in the kidney tissue of cisplatin group. In addition, the expression of Bcl-2 was significantly increased and the rate of positive expression of Bax was markedly reduced in cisplatin + maltol (50, 100 mg/kg) groups compared to cisplatin group in a dose-independent manner (Fig. 4A). Maltol pretreatment therefore alleviated cisplatin-induced apoptosis of renal cells.

### Maltol suppresses cisplatin-induced inflammation in renal tissue

The effects of maltol against cisplatin-induced inflammation in the renal tissues were evaluated by the expression of iNOS and COX-2 in renal tissue of mice by immunohistochemical staining (Fig. 4A). The results showed that the positive expression of iNOS and COX-2 were significantly increased in renal cortex and renal medullary in cisplatin treated mice. In contrast, pretreatment with maltol significantly suppressed the expression of iNOS and COX-2 in a dose-independent manner (*p* < 0.05). Furthermore, as summarized in Fig. 4B, compared to the normal group, the levels of TNF-α, IL-1β, iNOS and NF-κB in serum of cisplatin group were significantly elevated. In contrast, pretreatment of maltol with 50 and 100 mg/kg for 10 days significantly decreased the cisplatin-increased levels of inflammatory factors in a dose-independent manner except for NF-κB (Fig. 4B, *p* < 0.05).

### Protective Effect of maltol on the viability of Cisplatin-exposed HEK293 Cells

**Figure 5:**
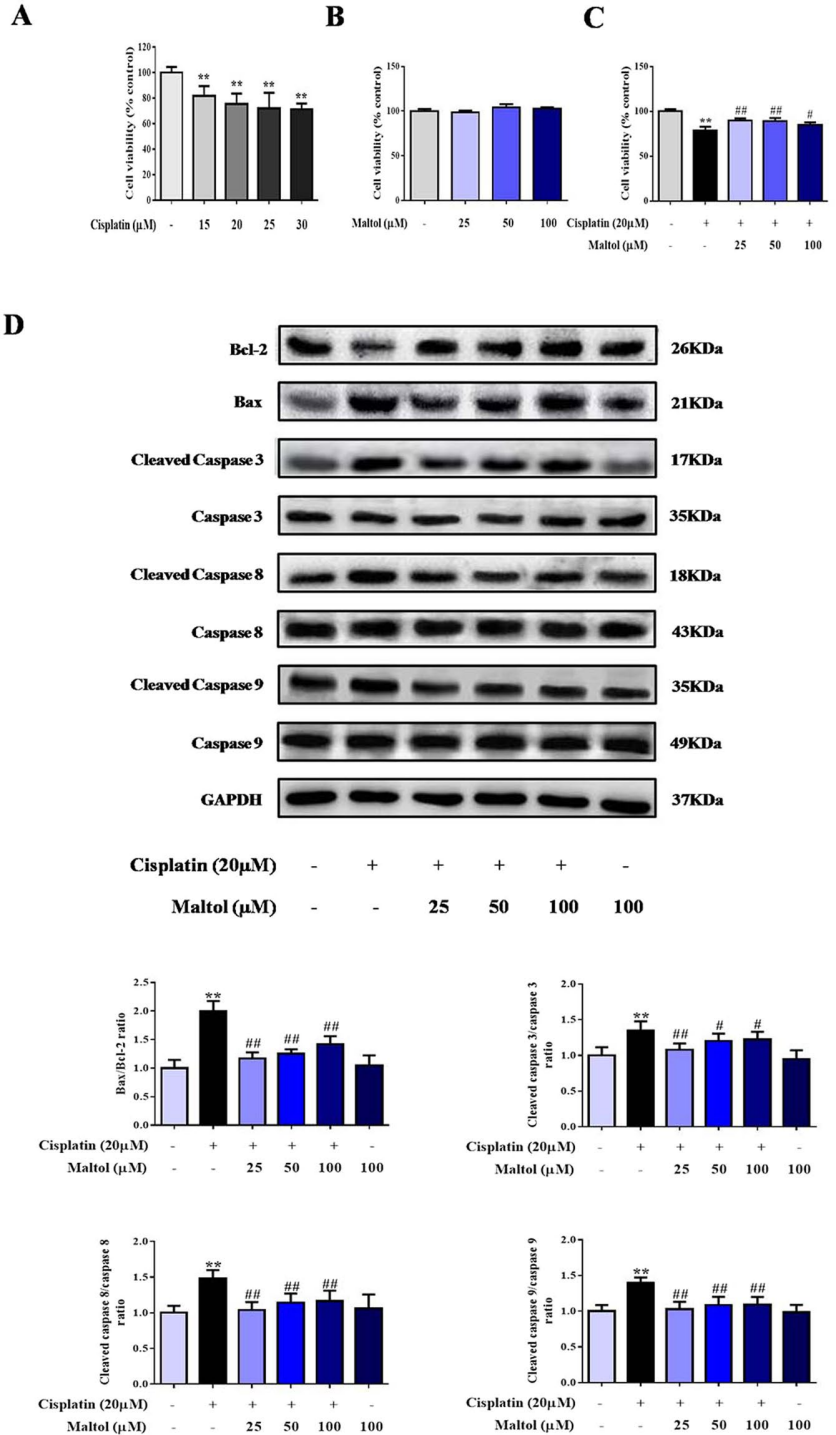
Protective effects of maltol on cisplatin-induced injury in HEK293 cells. (**A**) The cytotoxic effects of cisplatin on HEK293 cells. (**B**) Effect of maltol on the activity of normal cells. (**C**) The viability of HEK293 cells incubated with maltol after cisplatin exposure. Effects of maltol on the protein expression levels of Bcl-2, Bax and caspase 3, 8, 9 as well as GAPDH protein was used as a loading control. (**D**) Cells were used for western blot analysis of indicated proteins (upper panel). Column chart represents relative protein levels compared with the control group after normalization to GAPDH levels (lower panel) Values are expressed as mean ± S.D. *n* = 8. ^**^p < 0.01 *vs*. normal group; ^#^*p* < 0.05, ^##^*p* < 0.01 *vs*. cisplatin group.

Firstly, in order to determine the protective effect of maltol on the viability of cisplatin-exposed HEK293 cells, HEK293 cells were incubated with increasing concentrations of cisplatin for 24 h, then cell viability was determined by MTT assay. As shown in Fig. 5A, viability of HEK293 cells was decreased to about 70% with 20 µM cisplatin. To determine whether maltol caused directly cytotoxicity in cultured normal cells, HEK293 cells were incubated with different concentrations (25, 50, 100 µM) of maltol and cell viability was defined using MTT assay. As summarized in Fig. 5B, the results indicated that no significant cytotoxicity was observed in HEK293 cells treated by 25 to 100 µM maltol (Fig. 5B). In addition, maltol of various concentrations significantly improved HEK293 cells viability within 24 h after cisplatin incubation, which confirmed the protective effect of maltol on the viability of cisplatin-treated HEK293 cells (Fig. 5C).

### Maltol Ameliorates Cisplatin-Induced HEK293 Cells Apoptosis *in Vitro*

To further confirm the extent of apoptosis in HEK293, we measured the protein expression levels of Bax, Bcl-2, caspase 3, caspase 8, and caspase 9, which were all involved in apoptosis signal pathways by western blotting. As shown in Fig. 5D, cisplatin dramatically reduced protein expressions of Bcl-2 and increased protein expressions of Bax, caspase 3, caspase 8, and caspase 9. However, pretreatment with maltol for 24 h reversed the effect of cisplatin on the decreased expression of Bcl-2 and increased expression of Bax, caspase 3, caspase 8, and caspase 9 (Fig. 5D).

### Maltol regulated the AMPK/PI3K/Akt signaling pathways in HEK293 Cells

**Figure 6:**
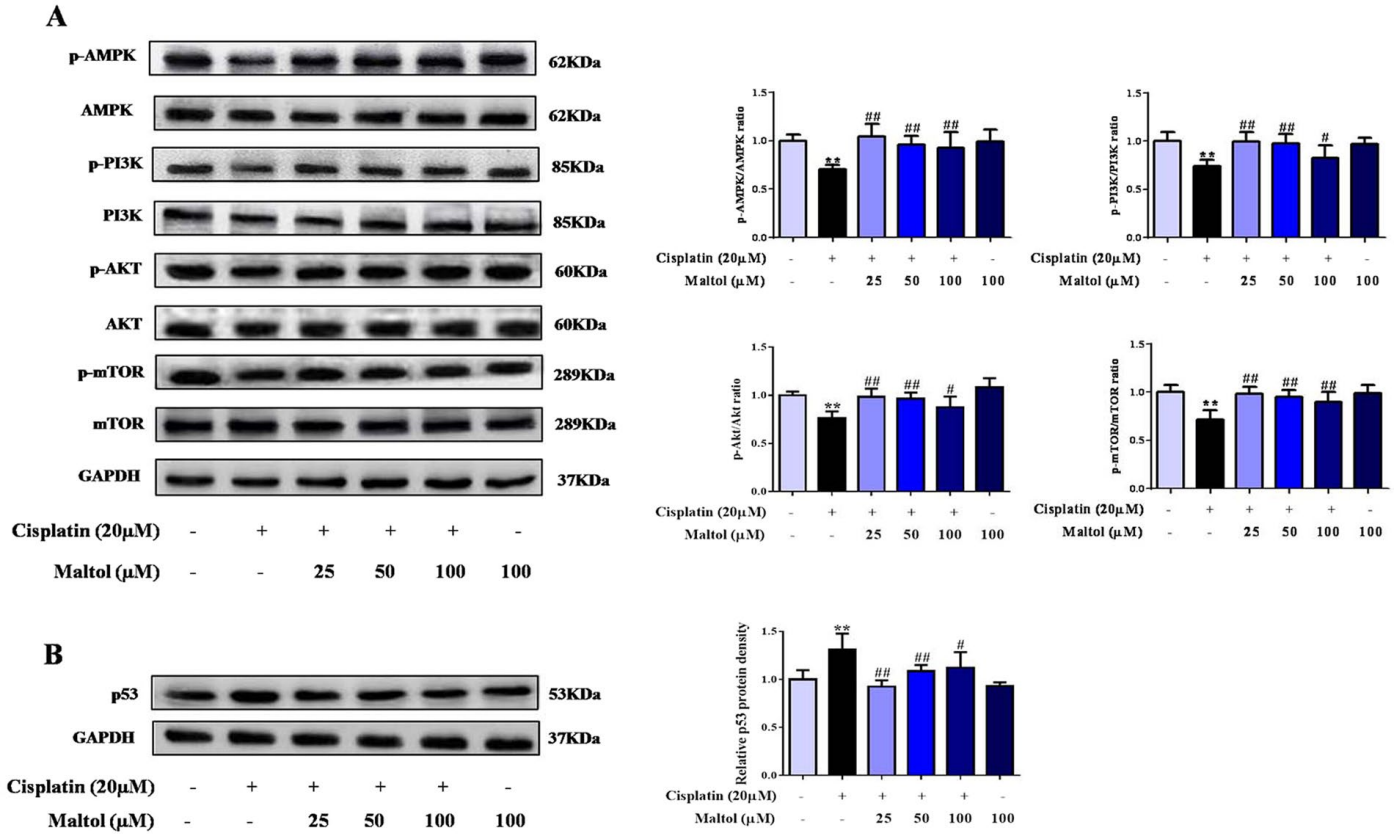
Effects of maltol on cisplatin-induced protein expression of PI3K/AKT signaling pathway. (**A**) Effects of maltol on the protein expression levels of AMPK/PI3K/Akt signaling pathway as well as GAPDH protein was used as a loading control. (**B**) Effects of maltol on the protein expression levels of p53 protein (left panel). Column charts show antibodies relative expression, protein expression was performed by quantification of relative protein expression analysis in HEK293 cells from each group (right panel). Values are expressed as mean ± S.D. *n* = 8. **p < 0.01 *vs*. normal group; ^#^*p* < 0.05, ^##^*p* < 0.01 *vs*. cisplatin group.

In order to investigate the protective mechanism of maltol pretreatment on cisplatin-induced acute kidney injury, the effects of maltol on Akt signaling pathway were investigated in HEK293 cells. Suppression of PI3K/Akt was responsible for renal injury and confirmed by a significant reduction in phosphor-PI3K and phosphor-Akt levers in HEK293 cells by western blotting analysis (Fig. 6). As summarized in Fig. 6A, expressions of p-AMPK, p-PI3K and p-Akt were decreased after cisplatin stimulation. Interestingly, maltol increased the expressions of p-AMPK, p-PI3K and p-Akt. These results suggested that maltol modulated apoptosis at least partly by acting on p-AMPK/p-PI3K/p-Akt signaling pathways.

To further study the effect of maltol *in vitro*, we next examined whether mammalian target of rapamycin (m-TOR), a central regulator closely related to protein synthesis and cell growth as well as a downstream signal of Akt, was involved in cisplatin pathogenesis (Fig. 6). As summarized in Fig. 6A, cisplatin significantly reduced mTOR phosphorylation in HEK293 cells; whereas maltol prevent the decrease in mTOR phosphorylation by cisplatin (Fig. 6A).

### Effect of maltol on cisplatin-evoked activation of p53 protein

As an important component of PI3K/Akt signaling pathway, p53 tumor suppressor protein plays a vital role in the regulation of various cellular stresses and cisplatin-evoked renal toxicity^32^. To identify whether the p53 was regulated by cisplatin exposure, the effect of p53 activation in HEK293 cells was examined. Cisplatin dramatically increased the expression of p53, whereas maltol reversed it (Fig. 6B).

## Discussion

Being widely used as antineoplastic, cisplatin therapeutic use is limited by its nephrotoxicity with about 25–35% of patients experiencing a significant decline of renal function after a single dose of cisplatin treatment^33,34^. Although cisplatin nephrotoxicity has been reported for many years, the protective effect of maltol on cisplatin-induced acute kidney injury and its molecular mechanism have not been demonstrated yet. In the present study, we investigated the potential protective effect of maltol against cisplatin-induced nephrotoxicity using mouse models and HEK293 cell models. Simultaneously, the possible mechanisms underlying the nephroprotective effect were explored by testing oxidative stress markers, inflammatory mediators as well as cell apoptosis. It has been demonstrated that maltol exerts an important protective effect on cisplatin-induced acute renal injury and the underlying molecular mechanisms may be through mitochondria-related AMPK/PI3K/Akt and p53 signaling pathways.

Numerous studies have confirmed that ROS-induced oxidative stress injury and activation of apoptotic signaling pathways are involved in the pathogenesis of acute kidney injury^6^. In this study, we identified the importance of oxidative stress in the improvement of cisplatin nephrotoxicity by maltol. Interestingly, these phenomena were effectively reversed by maltol pretreatment for ten days. In addition to oxidative stress, inflammation plays an important part in the pathogenesis of cisplatin-induced acute kidney injury and it is a complex phenomenon involving multiple cellular and molecular factors, which are rigorously controlled to avoid diseases^35^. TNF-α is a prototypical inflammatory mediator, which promotes cytokines generation and inflammation reaction by stimulating neutrophils and macrophages, ultimately resulting in cellular necrosis or apoptosis^36^. Similarly, IL-1β promotes the inflammation, causes fever, as well as stimulates the development and differentiation of the immune system^37^. In our previous work, maltol was reported to inhibit the overproduction of TNF-α and IL-1β in alcohol-induced liver injury in ICR mice^31^. In the present study, maltol effectively inhibited the secretion of these two cytokines. Thus, the inhibitory effect exhibited by maltol may contribute to the suppression the inflammation associated with excessive cisplatin-induced loss of renal function.

Previous studies have also confirmed that cisplatin-induced apoptosis through AMPK mediated signaling pathway is critical in the development of cisplatin-induced acute kidney injury^38^. Thus, cisplatin-induced kidney damage may be ameliorated via AMPK activation^17,18^. Furthermore, PI3K/Akt signal is a downstream target of AMPK and is closely related to cell growth and survival, highlighting the importance of Akt pathway as an attractive therapeutic target^39,40^. Recent evidence confirmed that the activation of PI3K/Akt was involved in kidney injury and was associated with the abundance in ROS production and apoptosis after cisplatin exposure^41^. Together with those reports, present results indicated that cisplatin exposure led to low expression of AMPK/PI3K/Akt *in vitro*, which was consistent with previous findings^17^. However, we demonstrated here that maltol pretreatment decreased AMPK and PI3K/Akt phosphorylation cascade activated by cisplatin.

Since Akt is regulated by its upstream PI3K and mTOR, mTOR is a central regulator that controls protein synthesis and cell growth^42,43^. We studied the effect of cisplatin on the activation of mTOR with results showing that maltol pretreatment decreased mTOR phosphorylation caused by cisplatin. This result clearly showed that cisplatin inhibited AMPK phosphorylation cascade, indicating clearly that the PI3K/Akt/mTOR were the potential drug targets for the prevention of cisplatin-induced acute kidney injury. AMPK may be a direct activator or positive regulator of PI3K/Akt, and the activated ROS suppress the pathway of AMPK; inhibiting the phosphorylation of its downstream substrate PI3K/Akt and mTOR. This study clearly indicated that the maltol pretreatment improved the inhibition of phosphorylation of PI3K/Akt, mTOR and the phosphorylation cascade of AMPK caused by cisplatin challenge. Maltol plays a favorable role in improving cisplatin-induced acute kidney injury.

**Figure 7:**
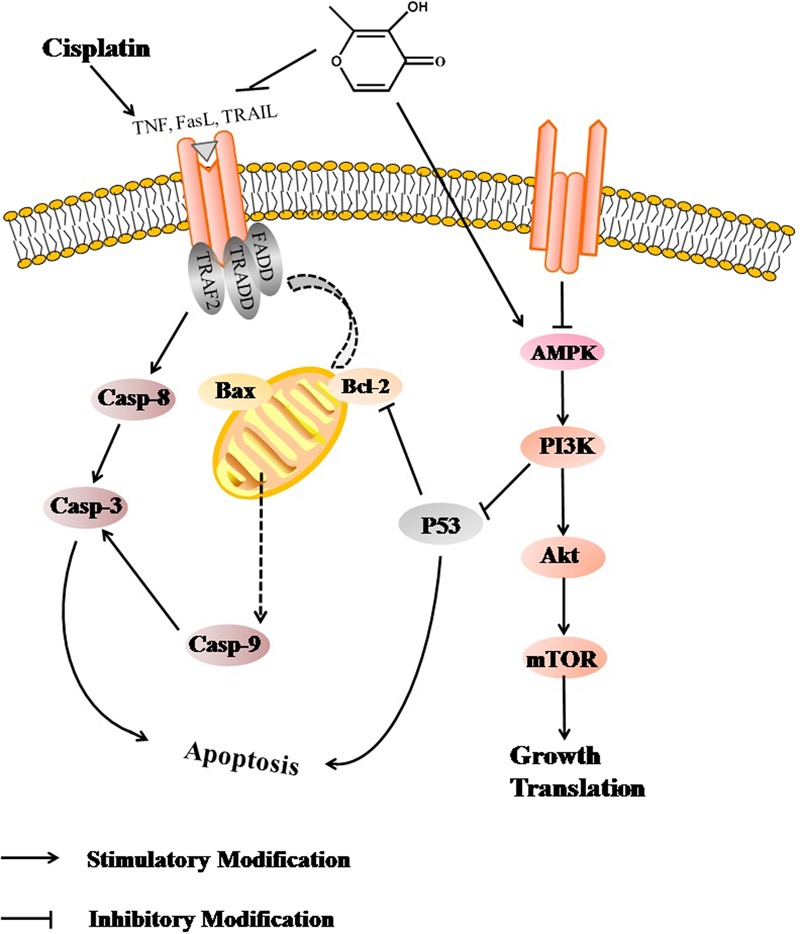
Scheme summarizing the inhibition of cisplatin-induced nephrotoxicity by maltol via the down-regulation of AMPK through PI3K/Akt pathway.

Apoptosis is a physiological process induced by various factors and is arranged through a variety of cell death signaling pathways^44^. Accumulated evidence has indicated that cisplatin-induced acute kidney injury was associated with cell apoptosis^12^. The Bcl-2 protein family determines the commitment of cells to apoptosis and activation of caspase-3 triggers the execution of cell apoptosis. Besides, p53 is also closely related to the apoptotic pathway. Many studies demonstrated that p53 functions as a downstream effector of PI3K/Akt to regulate the expression of anti-apoptotic protein Bcl-2^32,45^. It can not only up-regulate Bax expression, but also inhibit the anti-apoptotic effect of Bcl-2^46^. Cisplatin intoxication induced apoptosis and inhibition of the cell cycle in kidneys by increasing the expression of p53 and Bax and suppressing Bcl-2 expression; suggesting that inhibiting the p53 activation may be an important target to alleviate cisplatin nephrotoxicity^41^. Therefore, the expression of p53 protein was measured by western blotting analysis in this study. The results showed that the expression levels of p53 and Bax protein were significantly increased after cisplatin challenge, but this phenomenon was reversed by the pretreatment of maltol, consistent with the previous reports^41^. The findings in this work suggest that maltol may act as an anti- apoptosis effective agent through restoring the expression of Bcl-2 and inhibiting pro-caspase 3, 8, 9 cleavages. Taken all together, cisplatin induced renal tubular epithelial cell apoptosis through the decrease in the activity of AMPK, and the restrained expression of downstream PI3K/Akt signaling pathway. PI3K/Akt signaling pathway may restrain the activation of p53, which further facilitates the activation of Bax and caspase 3, caspase 8, caspase 9 to trigger apoptosis. Therefore, a more integrated mechanism of signaling pathway underlying maltol action against cisplatin nephrotoxicity should involve several key signal pathways (Fig. 7). Interestingly, the antitumor activity of maltol has been demonstrated. It may be speculated that maltol might have potential synergistic effect with cisplatin on antitumor activity^28,47,48^.

In summary, this study provides a new foresight into the potential molecular mechanisms of maltol action to protect cells against cisplatin-induced injury both in ICR mice and HEK293 cells. Maltol pretreatment markedly suppressed cisplatin-induced apoptosis, oxidative stress and mitochondrial dysfunction as well as the caspase 3 activation *via* the activation of AMPK/PI3K/Akt signal pathway. Administration of maltol or related compounds may be considered as a therapeutic strategy to prevent cisplatin-induced acute renal injury.
